# Italo-Swiss “Chalk and blackboard interactive 2-day workshop”—participants feedback

**DOI:** 10.1186/s13052-015-0164-0

**Published:** 2015-08-20

**Authors:** Pietro Camozzi, Pietro B. Faré, Camilla Lavagno, Gregorio P. Milani, Emilio F. Fossali, Mario G. Bianchetti, Sebastiano A. G. Lava

**Affiliations:** Pediatric Department of Southern Switzerland, Bellinzona, Switzerland; Foundation IRCCS Ca’ Granda, Ospedale Maggiore Policlinico, Pediatric Emergency Department, via della Commenda 9, 20122 Milan, Italy; Present appointment: University Children’s Hospital Bern, Bern, Switzerland

**Keywords:** Medical education, Problem-based-learning, Case-based learning, Residents

## Abstract

Ten “chalk and blackboard interactive workshops” have taken place between 2011 and 2015 in Southern Switzerland or Italy. Students, residents and expert pediatricians meet during 2 days and discuss 10–15 cases. Pediatricians promote reasoning, provide supporting information and correct statements. Emphasis is placed on history taking and examination, and on all participants being involved in a stimulating atmosphere. Thirty-seven participants were asked, ≥3 months after workshop-completion, to evaluate the workshop and a recent teaching session. Thirty answered and scored the workshop as excellent (*N* = 24) or above average (*N* = 6). The scores assigned to the workshop were higher (*P* < 0.001) than those assigned to the lecture-based teaching.

History taking and physical examination are the core elements of clinical reasoning and decision-making [1]. This process includes the identification and the interpretation of abnormal findings, the elaboration of hypotheses about the nature of the problem, the establishment of a working diagnosis, the selection of the crucial features, the exclusion of the diagnostic possibilities that fail to explain the findings, weighing the competing hypotheses and the choice of the most likely diagnosis [1]. Often, further history taking, supplemental examination and, lastly, laboratory or imaging studies are required to endorse or rule out the tentative diagnosis or to clarify which of the conceivable diagnoses is most probable. Obtaining, documenting and integrating history, physical and laboratory test findings into a meaningful diagnostic formulation represent a crucial issue of medical training. In our experience, lecture-based medical teaching regrettably emphasizes ancillary diagnostic studies and therapy over history taking, examination, clinical reasoning and decision-making. The “problem-based learning” approach rests on active learning in small groups, with clinical problems used as stimulus. Herein, the leader acts as facilitator, using expertise not to transmit facts but to provide reinforcement and guidance [2]. This learning approach generates enormous enthusiasm indeed [2].

Ten “chalk and blackboard interactive workshops” for future pediatricians and medical students have taken place between 2011 and 2015 either in Southern Switzerland (*N* = 5) or in Northern Italy (*N* = 5). For this purpose, a total of approximately 20–25 participants (6–8 medical students, 12–14 residents and 2–3 senior pediatricians; approximately half from Switzerland and half from Italy) meet during 2 days in a rustic hotel, where they ascertain a very interactive workshop for discussion of 10–15 real clinical cases from different fields of pediatrics. As opposed to problem-based-learning [1], expert pediatricians promote reasoning, provide supporting information such as clinical signs or symptoms, laboratory results and recent literature, and correct wrong statements if need be. Most of the time is spent to deepen and clarify basic clinical skills. For each case there are well-defined educational goals according to a pre-established lineup (Table 1) [3]. The presentation is dealt with using two blackboards, where relevant clinical data are noted. A projector is utilized exclusively for pictures of skin lesions and imaging or histological plates. During the “chalk and blackboard interactive 2-day workshop”, emphasis is placed more on understanding the role of history taking, physical examination and laboratory or imaging studies, and on all participants being involved and engaged in a stimulating atmosphere, than on obtaining diagnosis. Finally, the simple workshop structure, which is not subordinate to a rigid slide presentation file, may flexibly and agilely adjust to the different ability levels, personality characteristics, learning styles and cultural backgrounds of the participants [3].

**Table 1:** “Chalk and blackboard interactive 2-day workshop” tutorial

● Case-writing: a faculty and two learners (sometimes only one) select an appropriate and interesting case managed during their clinical practice, deepen their understanding on the topics chosen, develop different versions to cater the need of participants with different levels of preparation and define a clear set of learning objectives (time spent: 2–4 h).
● The learners and the faculty present the case, ask the participants to collect further history information and plan the physical examination.
● The participants make (and justify) an initial diagnostic assessment, suggest (and interpret) the laboratory tests and finally recommend management
● Treatment failure and possible complications are also discussed.
● The time spent to deepen history taking, examination, reasoning and decision making is considerably higher than that devoted to laboratory tests and therapeutic choices. The interpretation of diagnostic studies is also addressed (with emphasis on simple studies and potential errors).
● The total time spent for presentation and interactive elaboration of each case is 60 min or more.

Immediate post-session questionnaires frequently are used to accumulate feedback but the enthusiasm gained at educational events notoriously fades over time. To collect a more relevant feedback, participants from the penultimate (*N* = 7), last (*N* = 8) or both (*N* = 22) workshops were asked, approximately 3 months after workshop-completion, to evaluate the workshop and a recently attended lecture-based teaching session by means of an anonymous questionnaire. The general overall impression; the changes in motivation, confidence or abilities; the changes in knowledge and skills; and the changes in attitudes, beliefs and opinions were addressed on a five-point scale (1. very poor; 2. below average; 3. average; 4. above average; 5. excellent). Thirty (81 %) participants answered the questionnaire: five medical students (3 ♀ and 2 ♂ subjects, age 23–26 years), 20 residents (13 ♀ and 7 ♂ subjects, age 25–31 years) and five expert pediatricians (2 ♀ and 3 ♂ subjects, age 40–64 years). The median score of each filled in questionnaire was calculated and the Wilcoxon-paired-test used for comparison. The participants scored the “chalk and blackboard interactive workshop” as excellent (*N* = 24) or above average (*N* = 6). The scores assigned to the workshop were significantly higher (*P* < 0.001) than those assigned to the most recently attended lecture-based teaching (Fig. 1).

**Fig. 1:**
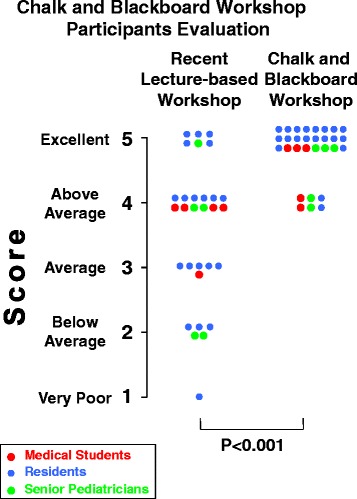
Five-point scale evaluation (1. very poor; 2. below average; 3. average; 4. above average; 5. excellent) of the “chalk and blackboard interactive workshop” and a recently attended lecture-based teaching session by 30 participants. The difference was statistically significant (*P* < 0.001)

In conclusion, the “chalk and blackboard interactive 2-day workshop” is a small-group teaching method. It combines theoretical knowledge with practical application and is more structured than “problem-based learning” (during sessions, there is no right or wrong answer, nonetheless identification of the proper solution is crucial at bedside). “Chalk and blackboard interactive workshop” method of learning and teaching resembles in part”case-based learning” [4], helps learners expand higher-order thinking ability and achieve deeper understanding of the to-be-learned content and appears to be very motivating and enjoyable. Obviously, our survey is based on attendee satisfaction, which does not demonstrate educational success of the intervention.
